# Counseling Aces: Experiences of Asexual Clients in China

**DOI:** 10.1007/s10508-025-03148-9

**Published:** 2025-05-23

**Authors:** Ruiqi Lu, Harold Chui

**Affiliations:** Department of Educational Psychology, Faculty of Education, The Chinese University of Hong Kong, Room 606, Chen Kou Bun Building, Hong Kong SAR, 999077 China

**Keywords:** Asexuality, Sexual orientation, Counseling experience, Multicultural competence, Interpretive phenomenological analysis, DSM-5

## Abstract

To understand Chinese asexual individuals’ mental health needs and service utilization, the present research aimed to explore Chinese asexual individuals’ navigation of their asexual identity within the Chinese sociocultural context and their experience of interacting with mental health practitioners on asexuality issues. A sample of 10 self-identified asexual individuals in China aged between 21 and 32 were recruited to participate in two semi-structured interviews. Interpretative phenomenological analysis was employed to explore the participants’ perspectives in depth. The analysis established four themes, including (1) identity exploration and negotiation, (2) service consideration, (3) satisfying experiences, and (4) unsatisfying experiences. Themes uncover the support needs of Chinese asexual individuals, their barriers and facilitators to help-seeking, and their interactions with mental health practitioners. Findings highlight the importance of incorporating cultural competence, cultural humility, and affirmative practice to assist asexual individuals’ navigation of identity and cultivation of resiliency. There is a pressing need to de-pathologize asexuality in mental health settings and integrate asexual issues in multicultural training and supervision.

## Introduction

Traces of asexuality’s presence in academic discourse date back to the nineteenth century, when sexologists observed individuals who lacked sexual feelings or desire (Winer, 2024c). Over time, these potentialities of asexuality were described and labeled in various ways. For instance, Kinsey et al. (1948) identified Group “X” to categorize individuals who reported no socio-sexual response. Storms (1979, 1980) was the first to explicitly introduce asexuality as a sexual orientation. Building on this, Bogaert’s (2004) influential work examined its prevalence and associated factors as a sexual orientation. Over the decades, scholarly interest in asexuality has grown, with researchers from various disciplines actively advancing this evolving field of inquiry.

Asexuality was characterized as the absence of sexual attraction to others in Bogaert’s (2004) work. However, as asexuality studies and online asexual communities continued to evolve, researchers have increasingly recognized the rich heterogeneity within the asexual population. Asexuality is now understood as a spectrum consisting of a range of sub-identities, including demisexuality and gray asexuality (Carrigan, 2011; Copulsky & Hammack, 2023; Nimbi et al., 2024). Given this diversity, an updated conceptualization defines asexuality as “both a spectrum and an umbrella term that generally refers to those who experience low/no sexual attraction” (Winer, 2024c).

Additionally, for asexual individuals, romantic and sexual attractions do not necessarily develop synchronously, and their sexual and romantic attractions/orientations are independent (Antonsen et al., 2020; Winer, 2024b). This further highlights the complexity and diversity of asexual experiences. In response, scholars have emphasized the value of an identity-based approach that centers on self-identification to better capture the nuances of asexuality and its diverse manifestations (Hinderliter, 2009; Scherrer, 2008). Researchers have increasingly examined asexuality as an identity category, exploring how individuals navigate their identities, construct meaning around their experiences, and negotiate various social challenges (MacNeela & Murphy, 2015; Mollet, 2020; Robbins et al., 2016; Winter-Gray & Hayfield, 2021).

### Challenges Faced by Asexual Individuals

One focal point in contemporary asexuality research concerns the “consequences” of identifying as asexual. When the construction of an asexual identity is a social and psychological process, the consequences of identification are also twofold (Scherrer, 2008). Firstly, from a sociological perspective, the emergence of the asexual community has made scholars aware of a series of societal norms regarding sexuality, including the assumption that sex is common and universal for all members of society by default (Gupta, 2015). These dominant societal norms, particularly compulsory sexuality, shape individuals’ lives in multiple aspects, including experiences of pathologization, social isolation, relationship difficulties, and dismissal of their self-identified asexuality by others (Gupta, 2015, 2017a). Under the influences of compulsory sexuality, asexual individuals experience significant personal and interpersonal challenges.

Combined with the erasure of asexuality from mainstream discourse and the limited availability of asexual knowledge, compulsory sexuality presents challenges for people to recognize asexuality as an option for identification (Winer et al., 2024). After finally arriving at an asexual identity, individuals find themselves positioned outside the social norms upheld by the majority, which can challenge one’s existing self-concept and stable identity (MacNeela & Murphy, 2015). The resulting sense of alienation can also negatively influence their self-worth (Brandley & Dehnert, 2024). Similar to other sexual minority individuals, asexual individuals report encountering negative reactions after coming out (Chasin, 2015; Cuthbert, 2022; Robbins et al., 2016). Examples of this include the denial of epistemic authority: After asexual individuals make their identity visible, people may deny the validity of their asexual identity by assuming that they have not met the right person or they are not yet sexually mature (Cuthbert, 2022; Gupta, 2017a). In terms of relationships, asexual individuals who reject traditional partnerships may face devaluation of their non-normative relationships by others (Chasin, 2015). Those in romantic relationships with non-asexual individuals may experience pressure from their partners (Gupta, 2017b), whereas those who pursue partnerships may encounter difficulties related to their asexuality (Van Houdenhove et al., 2015). These struggles that asexual people face when negotiating their identities highlight the need for professionals to understand how wider social norms can impact their lives, particularly in terms of identity development, identity validation, and relationship dynamics. Helping professionals who are aware of these challenges are better equipped to offer affirming and culturally competent support for asexual clients.

Secondly, from a psychological perspective, the unfriendly sociocultural environment adversely affects the general well-being of asexual individuals, leading to poorer mental health outcomes. As both culture and institutional systems privilege sexuality over asexuality (Bogaert, 2015), asexual individuals are subjected to biases, stereotypes, prejudices, and discrimination. The belief that “humans should be sexual” portrays asexual individuals as alien, robot, or monstrous, and harms them through dehumanization (Brandley & Dehnert, 2024). They are marginalized in social interactions, stigmatized as machine-like, medicalized as having sexual dysfunction, and discriminated against by heterosexual individuals (Bogaert, 2015; Chan & Leung, 2023; Chasin, 2015; MacInnis & Hodson, 2012; Przybylo, 2011). Moreover, although asexuality is recognized as one of the identities represented in the LGBTQIA+ umbrella, there is evidence of exclusion of asexual people within these spaces, which can be attributed to the misperception that they do not face oppression (Winer, 2024c).

Asexual individuals experience substantial levels of minority stress due to exposure to asexual-targeted biases in their social relationships, including interactions with family members, friends, co-workers, and healthcare providers (Chan & Leung, 2023). This contributes to negative mental health outcomes, such as greater risks of depression, anxiety, and other psychological symptoms, compared to non-asexual individuals (Borgogna et al., 2019; Yule et al., 2013). Asexual individuals may also internalize negative societal prejudices and stigmas about asexuality, a process known as internalized acephobia (Brozowski et al., 2025; Zheng & Su, 2022). Higher levels of internalized acephobia are associated with lower life satisfaction (Brozowski et al., 2025). These mental health needs of individuals who identify as asexual underscore the urgent need for professional support to address the challenges they face.

### Attending to Asexuality in Psychotherapy Contexts

Professional mental health practitioners play an essential role in providing culturally competent and affirmative care to asexual individuals. Counselors and therapists may become allies to asexual individuals and support them in further embracing their asexuality and flourishing in their social life (Pinto, 2014). However, while some asexual individuals reported having positive interactions and helpful experiences with practitioners, others still experienced interactions that further reinforced stigmas and stereotypes (Flanagan & Peters, 2020). Researchers summarized three main barriers for asexual individuals to access culturally safe mental health help: pathologization, microaggression, and practitioners’ lack of knowledge about asexuality (Schneckenburger et al., 2024).

Similar to other minority sexual orientations, asexuality has historical and theoretical connections with the healthcare systems (Foster & Scherrer, 2014; Scherrer, 2008). Historically, the absence of sexual desire has been seen as a diagnostic criterion for desire disorders, such as Hypoactive Sexual Desire Disorder (HSDD), leading to the medicalization and pathologization of asexuality. Scholars reviewing previous debates and existing research concluded that asexuality should not be classified as a mental health diagnosis, symptom of one, or sexual dysfunction (Brotto & Yule, 2017). Furthermore, labeling a variation in human sexuality as deviant fosters stigmatization (Bogaert, 2006). Both the academic and asexual communities have been striving to de-pathologize asexuality. However, despite the attempt to address the criticisms and concerns through the removal of HSDD, similar conditions still remain in the *Diagnostic and Statistical Manual of Mental Disorders*, now divided into two disorders specific to gender (Winer, 2024c). Asexual individuals continue to experience pathologization, which includes inappropriate diagnosis of asexuality as a mental, physical, or sexual disorder (Flanagan & Peters, 2020) and the harmful practice of sexual orientation change efforts by practitioners (Boot-Haury et al., 2024).

At times, biases and prejudices among practitioners may not manifest directly in pathological attitudes and behaviors but rather emerge in more subtle ways during therapeutic interactions (Shelton & Delgado-Romero, 2011), contributing to asexual individuals’ experiences of microaggressions. Providers frequently default to the assumption that asexual individuals are heterosexual, doubt clients who disclose as non-sexually active, or persuade asexual individuals to try sexual activities, thereby perpetuating harm rather than providing care (Schneckenburger et al., 2024). Asexual individuals reported practitioners’ disbelief in their asexual identities and found disclosing their identity with practitioners stressful and uncomfortable (Flanagan & Peters, 2020; Foster & Scherrer, 2014). Moreover, researchers have highlighted practitioners’ limited knowledge of asexuality as perceived by asexual clients, marking their knowledge level as “impoverished” and underscoring that many practitioners may not be able to provide quality care to asexual individuals (Foster & Scherrer, 2014, p. 428). Asexual clients have expressed the expectation that mental health practitioners enhance their knowledge of clients’ identities, as such understanding is necessary to provide inclusive and effective care (Flanagan & Peters, 2020).

The existing literature has contributed insights into how mental health practitioners may provide culturally competent service to asexual individuals, including gaining asexual-related knowledge, validating clients’ asexual identity, and displaying accepting and affirmative attitudes (Flanagan & Peters, 2020; Foster & Scherrer, 2014). However, the exclusive use of surveys in these two studies limited the depth of exploration into broader contextual factors, such as sociocultural dynamics that influence therapists’ interactions with asexual clients. As a result, there is a growing call for additional research in this area to aid practitioners in delivering culturally sensitive care and building positive therapeutic relationships with asexual clients. Moreover, current understandings of the issue are mainly based on research conducted in North America, with white samples predominating the participant pools, limiting the generalizability of research findings (Schneckenburger et al., 2024). Research on asexual clients in a new context, especially in a non-Western, non-English speaking country, can enrich professionals’ understanding of how this underrepresented population can be better assisted in mental health settings.

### Exploring Asexuality in Chinese Society

Scholarly discussions about compulsory sexuality have led to the examination of the concept of amatonormativity and its role in shaping asexual individuals’ lives (Glass, 2022; Vares, 2022). Whereas compulsory sexuality privileges sexuality, amatonormativity assumes that a “central, exclusive, amorous relationship” is normal for all humans (Brake, 2012, p. 88). Under the amatonormative paradigm, entering a committed heterosexual relationship, getting married, and having children are the only keys to happiness, regardless of one’s sexual and/or romantic orientation (Vares, 2022). Within the asexual spectrum, some individuals experience romantic attractions, whereas others may identify as aromantic, an umbrella term generally used for those who do not experience or experience low romantic attraction (Winer, 2024b). The experiences of intimacy and kinship are diverse, and some asexual individuals may not desire to conform to the traditional ideals of romantic relationships, marriage, and family (Tessler, 2023). They may be critical of the societal pressure to conform to amatonormativity, but at the same time, some of them may still desire the “happy family” it offers (Vares, 2022). Some find that society denies the validity of their non-normative intimacies (Glass, 2022), and some feel sad and lost due to being excluded from what is considered the key to happiness (Vares, 2022). Although the concept of amatonormativity has not yet been investigated in the Chinese context, researchers have uncovered how societal norms of prioritizing marriage and having children have influenced the lives of asexual individuals in China (Wong, 2015; Wong & Guo, 2020).

In Chinese society, marriage is seen as an obligatory duty and mandatory task that people must fulfill to conform to societal expectations (Hu & Wang, 2013). Under the influences of traditional Confucian culture, the family kinship system expects children to be filial and preserve the continuity of the family’s bloodline (Su & Zheng, 2022; Wen & Zheng, 2020). Parents holding deep beliefs around traditional culture tend to pressure lesbian, gay, and bisexual (LGB) individuals to enter conventional marriage and produce offspring (Hu & Wang, 2013). Evidenced by the Chinese saying: “There are many ways to be unfilial; the worst is not to have offspring” (Wong & Guo, 2020, p. 81), sexual minority individuals in China face immense pressure from these traditional values to have children and satisfy the role of a dutiful family member. Embracing a minority sexual identity in China risks abandoning social responsibilities, disappointing parents (Hu & Wang, 2013), and being condemned as unfilial. The existence of societal expectations, especially those posed based on marriage, filial, and reproduction norms, weaves a conflicting push–pull dilemma for some Chinese asexual individuals. Although the individuals feel a strong internal desire to embrace and express their asexual identities, the norms deeply rooted in Chinese culture may push them toward conforming to traditional relationships and family structures, such as getting married and reproducing.

The conflict between traditional culture and sexual identity ultimately becomes a stressor to individuals of minority sexual identity in China (Wen & Zheng, 2020). Research has shown that pressure to get married, rooted in traditional social norms, can contribute to depression, anxiety, and stress symptoms among Chinese LGB individuals (Zheng et al., 2020). When Chinese asexual individuals are trapped in a push–pull dilemma, it is reasonable to assume that they may also experience cultural-specific stress, which in turn negatively impacts their mental health. In China, asexual individuals reported poorer mental health compared to lesbian and gay individuals, experiencing higher levels of stress, anxiety, and depression (Zheng & Su, 2022). Quantitative data have discussed the psychological well-being of Chinese asexual individuals (Xu et al., 2023; Zheng & Su, 2022), yet further exploration is needed to understand their subjective experiences of mental health challenges. An expansion of knowledge on this issue can help identify areas requiring additional support, resources, or tailored interventions.

Despite a substantial need for clinicians and counselors to provide health services to asexual clients in China, barriers to their help-seeking still exist. These barriers can be grouped into practitioner-related and client-related factors. Practitioner-related barriers include a lack of LGBT affirmative training for counselors and therapists in China (Liu et al., 2023), limited access to service for Chinese sexual minority individuals due to staff shortage (Wang et al., 2021), and a lack of legal protections to prevent discrimination based on sexual orientation in service delivery (Suen & Chan, 2020). Client-related barriers, on the other hand, are influenced by the widespread mental health-related stigmas. These include public and self-stigma on mental illness (Xu et al., 2017), cultural-based concerns about help-seeking (Busiol, 2016; Shi et al., 2020), and negative attitudes toward mental health help and helping professionals (Shi et al., 2020). These stigmas exist beyond the immediate environment of psychotherapy sessions but can potentially influence clients’ engagement in the therapy process and their perception of therapy outcomes (Owen et al., 2013). To gain a more comprehensive understanding of asexuality issues in psychotherapy settings, the focus should extend beyond interactions within therapy sessions. It is essential to consider the entire help-seeking process, including asexual clients’ consideration of available services, their barriers and facilitators to access services, and their overall experiences with service use.

To fill the gap in empirical research on the mental health needs of asexual clients in China and their experiences of bringing up their asexuality to psychotherapy settings, the present research aims to explore (1) Chinese asexual clients’ experience navigating their asexuality within the Chinese sociocultural context and (2) their experience of seeking professional mental health help over asexuality issues.

## Method

The present study was an exploratory inquiry into asexual clients’ sense-making of their identity and help-seeking. The data collected for the study are experiential, involving the attempt to capture asexual individuals’ life and counseling experiences through their personal accounts. These characteristics make interpretive phenomenological analysis (IPA) a suitable approach to guide research design and analysis. As a participant-oriented method, IPA enables insights into healthcare services from the clients’ perspectives, thus making clients’ inner worlds accessible (Biggerstaff & Thompson, 2008). Utilizing the IPA approach in the current study can uncover the complexity of participants’ identity-related experiences, as well as their subjective reflections on their interactions with mental health practitioners.

### Participants

A total of 10 participants were recruited. Table 1 outlines participant characteristics relevant to the research questions. Among the participants, six were cisgender asexual women, and four were cisgender asexual men. Their age ranged from 21 to 32 years (*M* = 25.1, SD = 3.88), and the number of psychological counseling sessions they attended varied from three to approximately 24. More than half of the participants identified as experiencing heteroromantic attraction (*n* = 6); others identified as panromantic, biromantic, or questioning. Additionally, one participant identified as asexual with romantic attraction, without specifying gender direction. In terms of counseling services, four participants sought assistance from university counseling centers, and the remaining participants made appointments with various types of settings: private practice (*n* = 3), hospital (*n* = 1), online counseling platform (*n* = 1), and community counseling center (*n* = 1). The majority of counseling sessions took place in Mainland China (*n* = 9), with one participant received counseling in Hong Kong.

**Table 1:** Participants' demographic characteristics

Participant	Sex	Age (years)	Romantic orientation	Number of sessions	Type of settings
A	Male	23	Romantic	10	University counseling center
B	Female	32	Panromantic	3	Online counseling platform
C	Male	25	Heteroromantic	4	Private practice
D	Female	21	Heteroromantic	3	University counseling center
E	Female	25	Questioning	24*	Hospital
F	Female	23	Biromantic	4	University counseling center
G	Female	21	Heteroromantic	3	University counseling center
H	Male	22	Heteroromantic	3	Private practice
I	Female	32	Heteroromantic	15	Private practice
J	Male	27	Heteroromantic	4	Community counseling center

*Participant E could not recall the exact number of sessions attended but indicated that sessions occurred weekly over six months

### Researchers

The first author is a counseling trainee trained in the UK. She has completed a course in multicultural counseling during her studies and possesses a systematic understanding of qualitative research methods. Her interest in the research topic stemmed from a prior personal journey of exploring an identity within the asexual spectrum. The second author, a counseling psychologist trained in the USA, shares a keen interest in multicultural issues in counseling and supplements the team with his professional experiences working with clients of minority sexual identities. In his role as the academic supervisor of the first author, he played a vital role in conceptualization, research activity planning, data auditing, as well as the reviewing and editing processes. Throughout the research process, the authors collaborated in exchanging insights and negotiating diverse perspectives.

The research team embraces Heidegger’s stance toward phenomenology, which underscores the co-constructed nature of meaning-making and acknowledges that researchers bring their preconceptions to the interpretation process. Consequently, in IPA studies, data interpretation is inevitably shaped by the researchers’ preconceptions (Smith et al., 2022). These preconceptions serve as a valuable guide for interpretation. However, they risk reducing participants’ voice in the analysis, necessitating an awareness of potential researcher biases and their effects on the data analytic process.

Recognizing the potential impact of their own biases, the research team engaged in explicit discussions regarding their prior knowledge and experiences related to the topic under study. The first author reflected on her own positionality in relation to participants, acknowledging that her past identity exploration experiences can potentially result in preconceptions about participants’ experiences. Conversely, the second author stands in a relatively “outsider” position in relation to the asexual population. This less intimate position enabled him to see what the first author had taken for granted (Griffith, 1998), thus identifying some of the preconceptions that the first author could have brought to the study. Detailed strategies for eliminating researcher biases and enhancing the authenticity of interpretation are outlined in the section on analytic trustworthiness.

### Interview Protocol

IPA recommends one-on-one, semi-structured interviews to collect rich and detailed narratives from participants (Smith et al., 2022). The first author pre-defined questions and developed an interview protocol structured around two research questions (see Appendix for the interview protocol). The protocol consists of two separate interviews. The first interview aims to investigate how participants live with and make sense of their asexual identities. Key topics explored in this session included participants’ understanding of asexuality, their identity development, disclosure experiences, asexual community engagement, negotiation of intimacy and relationships, and their attitudes toward sexuality, marriage, and reproduction. Questions about participants’ demographic information were positioned at the end of this first interview.

The second interview aims to understand participants’ sense-making of their interactions with practitioners. This session began by focusing on pre-session dynamics, exploring participants’ motivations for seeking help, their counseling goals and expectations, rationale for choosing services and service providers, and past counseling experience. It then examined participants’ perception of counselors’ knowledge and attitudes regarding asexuality, helpful and unhelpful interactions, overall evaluation of counseling, and rationale for terminating or continuing sessions.

The second author reviewed the structure of the protocol, refined the core topics covered in both sessions to improve logical flow, and adjusted the wording of questions to enhance clarity and encourage richer responses. Two volunteers from the Chinese asexual community (i.e., the same population from which the study sample was drawn) were invited to participate in pilot interviews before finalizing the interview protocol. Responses from the pilot interviews enabled the research team to assess the appropriateness of interview questions. Feedback from the pilot interviewees was integrated into the final version of the interview protocol, and the two interviews were not included in the data analysis.

### Procedure

#### Participant Recruitment

Purposive and snowball sampling techniques were adopted to select experience-rich participants who can offer insights into the research focus (Smith et al., 2022). Participants were recruited through advertisements posted on two social media platforms: the Douban Asexual Forum and the Sina Weibo Asexual Super-topic. The former is the sister website of Asexual Visibility and Education Network (AVEN; Wong & Guo, 2020), and the latter is a microblogging social network platform in China equivalent to Twitter (X). The recruitment advertisement posted described the research aims, target participants, questions to be asked, compensation, the voluntary nature of the study, and encouraged potential participants to share the information with their friends and communities. Interested individuals were invited to fill in an online questionnaire that asked about their name, gender, age, sexual orientation, contact method, the number of counseling sessions attended in the past six months, and the type of settings that they attended. To obtain a reasonably homogeneous sample, as recommended when employing the IPA approach (Smith et al., 2022), recruitment criteria were established as (1) Chinese adult, (2) self-identified as asexual, (3) cisgender, and (4) attended more than three online video/face-to-face psychological counseling sessions with a certified counselor in China within the past six months.

The online questionnaire received a total of 38 responses. Nine of these responses did not meet the inclusion criteria, and an additional six were identified as repeated submissions. Subsequently, the researcher contacted 23 potential participants via instant messaging apps (QQ or WeChat). Three of these attempts were unsuccessful due to the inability to locate the account users, and 17 respondents expressed interest in learning more about the study upon receiving the researcher's message. The researcher then assessed their eligibility and provided them with a Chinese version of the interview protocol for reference. After reviewing the interview protocol, 10 eligible participants opted to participate in the study and formally consented by signing the consent form.

#### Interview and Transcription

Participants were invited to engage in two semi-structured interviews, all of which were conducted online via QQ or WeChat voice calls (similar to WhatsApp voice calls). The interviews were conducted in Chinese, which was the first language for both the interviewer and participants. The first interview, focused on asexuality, lasted approximately 45 min to an hour (*M* = 49.58 min, *SD* = 11.92). Following the first interview, participants were asked about their availability for a second interview. The second interviews were then scheduled according to their availability, and all were conducted within ten days (*M* = 4.0 days, *SD* = 3.6). The second interview, centered on experiences with psychological counseling, lasted between 30 and 45 min (*M* = 39.52 min, *SD* = 9.34). After completing the two interviews, participants were compensated with 100 RMB (~ 14 USD) as an appreciation for their participation. All interviews were audio-recorded by an electronic voice recording device. The first author wrote down keywords and observations during interviews, supplementing the data analysis. The interview recordings were transcribed verbatim to capture all words and notable interactions in the conversations (Smith et al., 2022). Any identifiable information belonging to the participants was removed from the transcript, and identification codes were assigned to each transcript.

### Data Coding and Analysis

The present research employed the IPA analytic approach recommended by Smith et al. (2022), adhering to the use of new terminologies. Notably, the authors proposed a terminology shift from “emergent themes” to “experiential statements.” The analysis of each case began with the researcher immersing themselves in the interview transcripts through multiple readings and reviewing interview notes. Upon gaining a comprehensive understanding of the texts, the researcher began writing exploratory notes to interpret the participant’s feelings, thoughts, and words. These exploratory notes were written in the same language (Chinese) used by the participants to recall their experiences, facilitating the reconstruction of “the original experiences in its own terms” (Smith et al., 2022, p. 30).

Moving to experiential statement construction, IPA requires a shift in perspective from participants to researchers. This transition was facilitated by introducing a layer of translation between two analytic steps. The exploratory notes written in Chinese were translated into English with contextual comparisons made between the two languages (Suh et al., 2009). Cultural-specific words were retained during translation to preserve the original meanings conveyed by the participants. Subsequently, the researcher constructed experiential statements in English by summarizing key focal points of the exploratory notes, ensuring each statement was concise yet rich in detail. The Personal Experiential Themes (PETs) were generated based on manual methods, with the researcher writing individual experiential statements on separate pieces of paper. The researcher then re-evaluated each statement, searched for interconnections across them, and clustered them into a participant’s PETs table. This process was repeated for each participant’s transcript. Once the PETS for all ten transcripts were completed, the researcher searched for patterns of similarities and differences across PETs to create a set of Group Experiential Themes (GETs).

#### Sample Size

Qualitative research requires a clear rationale for sample size decisions (Dworkin, 2024). Although saturation is a common reference point, the research team did not emphasize it in this study for two concerns. First, saturation is challenging to apply in IPA studies, given that each participant’s unique and rich life accounts offer the potential for ongoing insights (O’reilly & Parker, 2013; Wray et al., 2007). Considering the iterative nature of analysis, theme emergence can be endless (Brocki & Wearden, 2006; Green & Thorogood, 2018). Second, IPA prioritizes the evolving key elements within each individual’s account rather than the identification of repetitive themes across participants (Saunders et al., 2018).

Instead of saturation, the present study adopted Malterud et al.’s (2016) concept of “information power,” which takes into account five factors that justify the sample size. First, while the two research objectives are moderately broad, the focus on an underrepresented group, Chinese asexual clients, narrows the scope of the study, allowing for a more focused analysis within a smaller sample size. Second, the sample was carefully selected based on specific criteria, including participants’ help-seeking experience primarily related to asexuality and their attendance at at least three counseling sessions, which supports the richness of their accounts. Third, the interviews were guided by a semi-structured interview protocol grounded in established knowledge and existing frameworks related to sexual minority identity, sociocultural contexts in China, and help-seeking behavior, helping to ensure that research data are interpretable through relevant concepts and models.

Additionally, the quality of discussion was enhanced through two rounds of interviews, allowing for a deeper exploration of each research question. Prior interview training from an experienced supervisor and the use of open-ended follow-up questions also aimed to increase the quality of communication between the researcher and the participant. Finally, IPA involves the examination of varied perspectives within the group by analyzing both the patterns of convergence and divergence (Smith et al., 2022). In the iterative process of data collection, transcription, and analysis, the research team noticed that the analysis of the data from the tenth participant contributed to a satisfactory level of depth and breadth to each key topic covered in the interview protocol. The evaluation of the recurrence and diversity of themes across participants led the research team to determine that ten participants (20 interviews) were a manageable and adequate sample size for generating shared group-level themes while still capturing distinct voices.

#### Analytical Trustworthiness

The present research utilized multiple methods outlined by Lincoln and Guba (1985) to enhance trustworthiness, including peer debriefing, member checking, and data auditing. Firstly, the first author conducted a peer debriefing session to expose her analytic process and outcome to impartial peers outside the study. External feedback gained from the sessions helped identify missing themes early in the analysis stage. Secondly, the research team adopted the basic level of member checking. All participants were invited to review their respective transcripts for clarification and potential amendment. Among them, eight participants did not provide any corrections, whereas two participants offered clarifications to adjust the language or tone of their responses to better convey their intended meaning. However, the research team chose not to ask participants to review the interpretations of the data. This decision was made based on the recognition that interpretations are context dependent, and participants’ perspectives on the same topic may evolve during the time lag between data collection and analysis (Varpio et al., 2017).

Thirdly, data auditing was employed to enhance the coherence and credibility of interpretations (Lincoln & Guba, 1985; Smith et al., 2022). The second author served as the auditor, having access to the audit trail including the research proposal, interview recordings, transcripts, exploratory notes, experiential statements, PETs, GET, and the final write-up. The second author assessed the quality of statements, considered alternative expressions, and suggested additional insights to enrich the analysis. Importantly, the second author assessed whether the first author’s interpretations were grounded in the data or potentially influenced by preconceptions. Finally, the research team discussed to reach a consensus on interpretations before finalizing the analysis results.

## Results

The Group Table of Experiential Statements generated four themes: identity exploration and negotiation, service consideration, satisfying experiences, and unsatisfying experiences. The four themes encompassed a total of ten subthemes, which are further detailed below. Table 2 displays the frequencies of themes and subthemes. The interview guide outlined that participants select experiences with only one practitioner to share. However, two participants (E and J) provided accounts involving a second practitioner to illustrate both helpful and unhelpful interactions from their perspectives. Given that these experiences align with patterns identified in other participants’ accounts, the authors integrated their PETs into the broader illustrations. The subsequent section presents the themes and subthemes, accompanied by representative quotes.

**Table 2:** Qualitative results of Chinese asexual clients’ counseling experiences

Group experiential themes and subthemes		Frequency, *n* (%)
*I. Clients’ identity exploration and negotiation*		
A. Identity exploration: finding a path in the fog	10 (100)	
B. Identity negotiation: challenges and opportunities coexist	10 (100)	
*II. Clients’ consideration of service*		
A. Seeking help with identity exploration and negotiation	10 (100)	
B. Positive attitudes and past experiences facilitate help-seeking	7 (70)	
C. Tailoring services to individual needs and preferences	6 (60)	
*III. Clients’ satisfying experiences in counseling*		
A. Culturally informed practices enhance communication	5 (50)	
B. Positive counselor characteristics strengthen alliance	4 (40)	
C. Helpful counselor activities contribute to effectiveness	6 (60)	
*IV. Clients’ unsatisfying experiences in counseling*		
A. Lack of culturally informed practices impairs relationship	4 (40)	
B. Lack of helpful counselor activities hinder effectiveness	3 (30)	

### Theme 1: Client’s Identity Exploration and Negotiation

#### Identity Exploration: Finding a Path in the Fog

Participants commonly described that their identity exploration began with the recognition of being different from others, which has led to feelings of discomfort and confusion. During the process of exploration, five participants questioned whether they were same-sex attracted, or contemplated the possibility of experiencing sexual dysfunction. Participant A (male, romantic, aged 23) said, “This thing (unable to feel sexual attraction) actually caused me some distress, and it has also led me to wonder whether I might be gay…I actually found myself trapped in a state of confusion about who I am.”

Before arriving at an asexual identity, participants tended to utilize various approaches to facilitate identity exploration. This included searching for keywords to describe their own characteristics on the internet, reading psychology-related books, watching sexual minority-inclusive television, and engaging in online communities. Online asexual communities in China appeared to be one of the common platforms for respondents to explore their identity and connect with other asexual individuals. Some participants even accessed foreign online asexual communities to browse educational content and connect with international members. However, participant C (male, heteroromantic, aged 25) noted that the significant gap between online and offline experiences fostered a sense of alienation in real life, contributing to self-doubt:

You gradually realize that only in certain communities do you find like-minded peers. People in your daily life are not like this, creating a significant contrast. It gives you a sense of not quite fitting in with those around you, and that there might be something wrong with yourself.

Eight participants highlighted the exciting moment when they realized that their personal experiences resonated with the concept of asexuality. Participant E (female, questioning, aged 25) vividly described this realization, stating, “It’s like you’ve been trying to find a path out of a fog, bumping around everywhere, and then suddenly, everything seems to be lighted up.”

#### Identity Negotiation: Challenges and Opportunities Coexist

Coming to an asexual identity merely marked the beginning of narratives. For all participants, asexual identity appeared to be a double-edged sword. On one hand, the existence of an identity that fits oneself can facilitate self-acceptance and validation. As participant A (male, romantic, aged 23) stated, “It allowed me to better accept myself, and led me to find a sense of belonging…My past experiences, thoughts, and feelings could finally be reconciled.” On the other hand, holding an identity that conflicts with the socially expected norms of sexuality evoked feelings of deviance and abnormality. Four respondents shared that these feelings have pressured them to “fit in” with the majority population, even though the process is uncomfortable. Participant B (female, panromantic, aged 32) concluded, “Many asexual individuals actually feel quite twisted and suppressed internally. They are afraid of (being different), yet unwilling and unable to conform to the heterosexual norms.”

Deciding on whether or not to disclose one’s identity to others is another daunting task. Participants generally perceived a need to disclose their asexual identity, particularly to friends, parents, and partners. However, the anticipated negative reactions from others and the negative impacts after disclosure led some of them to withhold their identity. Participant H (male, heteroromantic, aged 22) said, “Would discussing this with others make them feel repulsed towards me? And also, if my identity is exposed, will it bring troubles into my life?” For those who disclosed to at least one person, five received support and understanding, acknowledging that disclosure had a positive impact on their relationships. Conversely, four encountered disbelief, dismissal, and denial, leading to negative emotions and discouragement from future disclosures.

Participants have been considering how to accommodate their asexual identities within romantic relationships. All respondents in this study identified as romantic-oriented and expressed a desire for romance, intimacy, and relationships that emphasized qualities beyond sexuality. Nevertheless, they frequently voiced worries that asexuality may pose challenges to the establishment and maintenance of relationships. As participant D (female, heteroromantic, aged 21) said, “I’m concerned that if I have no sexual desires, it might affect my future romantic relationships. As I become less desirous, I might even lose the desire to pursue romantic relationships altogether.”

The preference not to be involved in sexual behavior with others has left most participants anxious about marriage and reproduction, albeit for differing reasons. Those leaning toward marriage and reproduction saw their asexuality as a major challenge. In contrast, those who did not plan to marry or have children found meeting societal expectations a more significant hurdle. Moreover, the influence of family pressure and parental expectations was particularly salient in these narratives. Five participants stated that pressure from the family had influenced their decisions to get married and have children, and four described the dilemma in which their parents’ opinions conflicted with their own life plans. Participant H (male, heteroromantic, aged 22) stated, “Parents tend to like, pressure you into marriage, you know, given they’re from an older generation…Their thoughts will definitely be influenced by the outdated ideas typical of their time, which will always hang over us.”

### Theme 2: Client’s Consideration of Service

#### Seeking Help with Identity Exploration and Negotiation

Participants recognized the necessity of seeking professional support at various stages of their journey in exploring and negotiating their identity. Three participants sought help during the identity search process to explore who they are and learn more about asexuality. Four participants sought counseling support after having identified themselves as asexual to deal with the experience of perceived deviancy and uncertainty regarding whether asexuality constituted psychological issues. For example, Participant I (female, heteroromantic, aged 32) said, “I felt I was abnormal at first, and my mind was occupied with the feelings of being different from the group, so I couldn’t quite accept it… I felt like I am ill.”

The remaining three respondents struggled with challenges throughout their process of identity negotiation, which included difficulties in disclosing their identity, navigating asexuality within relationships, and meeting societal expectations.

#### Attitudes Toward Counseling Influence Help-Seeking

Participants expressed that their previous positive help-seeking experiences and positive attitudes toward professional mental health help made them more willing to consider services. In contrast, some respondents discussed how stereotypes toward mental health services served as a barrier for asexual individuals to seek help. Participant F (female, biromantic, aged 23) said:

Many people tend to have stereotypical impressions, thinking that only people with mental illnesses do such things, right? If you suggest that they go for counseling…that makes them feel like if they do so, they’re admitting they have problems themselves.

#### Concern About Confidentiality and Risk of Pathologization

Participants weighed various factors when faced with available service options. Four of them emphasized that due to the sensitive nature of asexuality topics, confidentiality was the primary concern. The potential risk of personal information leakage in school-based services and the perceived lack of privacy protection in hospital-based services hindered their willingness to disclose fully. Some tended to select services that could grant them a greater sense of confidentiality and anonymity, even if accessing these services was less convenient.

The historical classification of asexuality as a desire disorder has made pathologization another main concern. Three participants expressed a preference for therapists over psychiatrists to avoid being pathologized. Participant I (female, heteroromantic, aged 32) recounted a previous experience with a psychiatrist: “You could sense in his eyes that he viewed you as ill, and he interacted with the kind of peculiar look or attitude. In their medical definition, this is deemed an illness; he believes you are ill.”

### Theme 3: Satisfying Experiences in Counseling

#### Culturally Informed Practices Enhance Communication

*Cultural Knowledge.* Participants commonly anticipated the necessity of explaining asexuality to the counselor early on in sessions. Two of them, however, were surprised when they discovered that their counselors were already familiar with the concept. They highlighted that the counselor’s demonstration of knowledge about asexuality encouraged them to engage in further discussion. Participant H (male, heteroromantic, aged 22) said, “I felt like… I’ve found someone I could confide in, someone with whom I could completely open up and pour out anything, like expressing all the thoughts in my heart and mind, as if I am confiding everything to her.”

*Openness to Learn.* As anticipated, the majority of participants found themselves in situations where their counselors were unfamiliar with asexuality. However, three participants noted that the counselors’ openness to knowing more despite their initial lack of knowledge signaled respect and acceptance. Participant E (female, questioning, aged 25) noted:

Because I feel that being asexual is not about whether others can truly agree with you. It’s about mutual respect. You confide in me about these things, and I am genuinely listening, trying to understand you—I think being able to adopt this stance is already very good.

*Affirmative Practice.* Counselors’ affirmative response to participants’ disclosure of their asexual identity has instilled in them a sense of formal recognition and validation from a professional. As Participant B (female, panromantic, aged 32) noted, “When I truly felt who I was, and then excitedly shared with him, he also recognized it…it felt like getting recognition from a professional.” Four participants conveyed that their counselor acknowledged their journey of self-discovery, encouraged them to embrace their asexual identity, and affirmed their asexuality. The counselor’s affirmative approach has further facilitated participants’ engagement. Participant D (female, heteroromantic, aged 25) recalled the counselor’s response after her identity disclosure:

She said that I’m actually bold in expressing myself and facing myself, and she recognized (my disclosure) this way. At that moment, I felt a deep sense of peace within, which encouraged me to open up about my inner world, enabling me to have even deeper conversations with her.

*Cultural Sensitivity.* The counselor’s culturally sensitive approach was seen as pivotal for facilitating comfortable interactions. Two participants noted that the counselor’s use of gender-neutral language during interactions made them feel respected. Participant A (male, romantic, aged 23) said:

My counselor was the first to use the term “partner”... It was a very accurate description. She didn’t ask me for specifics about my partner’s information. When she referred to that term, I felt it was a non-offensive and accepting state.

Moreover, three participants shared how their counselors interacted with them in a respectful and non-intrusive manner. The counselors refrained from prying into their asexuality or asking intrusive questions, which fostered a sense of comfort during communication. Participant H (male, heteroromantic, aged 22) said:

Throughout our conversation, she never once asked me why I am this kind of person, or why I have this psychological makeup...This made me feel less guarded or doubtful about her, and it was very comforting. I felt I could share anything with her.

#### Positive Counselor Characteristics Strengthen Alliance

*Non-judgmental.* Four participants remarked on their counselor’s non-judgmental attitude toward their asexuality, emphasizing how it conveyed a sense of acceptance. Participant A (male, romantic, aged 23) said, “I felt the counselor did not have a stance (toward asexuality). Yes. It was like the counselor being accepting of every emotion that arises naturally in you.” Participant E (female, questioning, aged 25) vividly recounted the interaction with the counselor and expressed a profound sense of relief, attributing it to the counselor’s non-judgmental listening:

When you talked (about being asexual) to her, it was just like telling her, “Let’s go out for dinner tonight,” and you think of a particular restaurant you’d like to try...and she goes along with your choice. Afterwards it became such a normal and common thing, I felt that my worries just faded away.

*Empathy.* When recounting memorable therapeutic moments, three participants expressed gratitude for the counselor's empathetic approach to understanding their concerns. Participant B (female, panromantic, aged 32) explicitly highlighted the counselor’s empathetic understanding of asexuality, stating:

He possessed empathy. He could, during times when I was greatly confused or felt that nobody understood me, stand in my shoes, using my thoughts, ideas, and feelings to provide me with guidance... I am very grateful for having someone who can truly understand me.

#### Helpful Counselor Activities Contribute to Effectiveness

*Facilitating Identity Exploration.* Participants reported a diverse array of helpful activities that their counselor engaged in during sessions. Six participants specifically mentioned how their counselor supported them in exploring, understanding, and accepting their asexual identity. For instance, When Participant J (male, heteroromantic, aged 27) expressed doubts about whether asexuality could be considered a psychological issue, the counselor offered insights to assist him in depathologizing asexuality. As Participant J described:

She said that everyone has their own characteristics. This (being an asexual individual) cannot be used as a basis for saying that you have psychological issues, but rather it can only be seen as a personal characteristic or orientation of yours.

*Facilitating Identity Negotiation.* Six participants reported discussing with the counselor the challenges they encountered in romantic relationships due to their asexuality, and they found the counselor’s responses to be helpful. Participant J (male, heteroromantic, aged 27) particularly emphasized the counselor’s effective guidance regarding entering romantic relationships. He said, “I feel that it indeed addressed my needs, including a problem that might have been bothering me deep down. It feels like a sudden enlightenment.” Participant A (male, romantic, aged 23) reacted positively when he heard about the counselor’s self-disclosure: “So I still found it quite fascinating. She shared with me that she has been with her partner for many years, but they also argued and got upset about certain things.”

Two participants noted that their counselor raised their awareness about the impact of social norms and traditional beliefs on their process of identity negotiation. Participant B (female, panromantic, aged 32) described how the counselor assisted her in understanding that her internal conflict arose from the tension between her asexuality and traditional beliefs: “He said that the various, excessive inner struggles that I experienced were related to my desire to pursue traditional norms, but deep down inside, I am actually in great torment. Yes.”

Participant I (female, heteroromantic, aged 32) shared how discussing the role of societal norms with the counselor helped her to de-pathologize asexuality. She commented, “I feel like I am taking a more positive, healthier approach now. This is simply an issue that most people are not familiar with or cannot accept, but it doesn’t mean I’m ill.” In addition, participants listed helpful suggestions from their counselors, including recommendations to connect with online asexual communities, develop social support networks, cultivate individually meaningful hobbies, and acquire self-healing techniques to cope with negative feelings. Participant H (male, heteroromantic, aged 22) described: “So, if faced with various difficulties in the future, particularly psychological ones, I can find someone to confide in or discover my own approaches to rediscovering the beauty in the world.”

### Theme 4: Unsatisfying Experiences in Counseling

#### Lack of Culturally Informed Practices Impairs Relationship

*Lack of Cultural Knowledge.* Three participants observed that their counselor’s unfamiliarity with asexuality resulted in misinterpretations of their identity. Although Participant B (female, panromantic, aged 32) found her overall counseling experience helpful, she also experienced these misinterpretations. She said, “He basically thought that I was frigid… I could sense that he believed my psychological aversion (to sex) was due to some past trauma, but I feel like I’ve always been averse.”

Participant G (female, heteroromantic, aged 21) noticed that the counselor’s knowledge gap has led to avoidance of asexuality-related topics. This participant further discussed the sense of isolation originating from the counselor’s dismissive attitude. She said, “She just sat beside me, and I didn’t feel that she was engaging in close and attentive listening. Sometimes there’s a sense of isolation. It’s like she’s listening to what I’m saying, but she doesn’t fully understand or empathize.” Participants tended to link the counselor’s lack of knowledge about asexuality to the concept’s invisibility in China. This includes its limited public awareness and the scarcity of related research and information accessible to counselors.

*Lack of Openness to Learn.* Two participants expressed feelings of disappointment due to their counselor’s unwillingness to learn about asexuality. Participant E (female, questioning, aged 25) contrasted the responses of different counselors when she disclosed her asexual identity:

She said it wasn’t her area of expertise, and she couldn’t help much, but she respected me. However, her response made me feel a bit disappointed and cold. When I spoke to the second counselor... she also didn’t have a deep understanding, but she asked me to educate her, and she listened patiently.

Moreover, the counselor’s lack of interest in further understanding participants’ asexuality discouraged them from sharing more. Participant G (female, heteroromantic, aged 21) described:

If she didn’t understand (asexuality), she could ask more questions; I didn’t mind because I am willing to share my feelings. However, after I finished sharing…it seemed like she didn’t want to delve deeper. So my choice was, since she didn’t seem interested in discussing further, I didn’t have much to say either.

*Lack of Cultural Sensitivity.* Two participants expressed that the counselor’s lack of sensitivity to their unique circumstances brought them negative impressions. They perceived the counselors’ responses to be based on general guidelines rather than a personalized understanding of their specific situations. Participant J (male, heteroromantic, aged 27) mentioned his previous, unhelpful experiences with another counselor. He said, “It’s more like if someone needs cold medicine, and there are a total of ten cold medicines, then I’ll just package them all together and give them. This way, they might recover faster.”

Furthermore, the counselor’s deficiency in cultural sensitivity might lead to misguided advice. Participant J talked about the counselor’s “kind reminders” on the consequences of being asexual:

She said that in my life, I should be as cautious as possible and try to conceal myself better... She said that I would be less likely to be hurt by others this way. Actually, I do not agree with this statement. I feel that her point is too absolute.

#### Lack of Helpful Counselor Activities Hinder Effectiveness

*Interpersonal Issues.* Misalignment between the participants’ expectations in help-seeking and the assistance offered by the counselor resulted in a perceived lack of counseling effectiveness. Two participants expressed that the counselors’ passive listening and occasional confrontation rather than providing guidance led them to perceive the session as ineffective. Additionally, one participant noted that the counselor’s advice to make a breakthrough toward self-acceptance was unhelpful because it is challenging to implement. Participant C (male, heteroromantic, aged 25) said:

The usual words of guidance didn’t seem particularly helpful or effective. I thought of making a breakthrough by disclosing this (asexuality) to my family; I thought that this would be a good starting point... However, I still don’t feel determined to do it.

*Microaggressions.* Participants’ narratives revealed two incidents of microaggressions. Participant J (male, heteroromantic, aged 27) recounted feeling disrespected during his interactions with his previous counselor. He said, “I felt like during our conversations, she was constantly speculating about me, always trying to pinpoint some sort of issue in me…it was more likely that she saw me as an ill person, without showing much respect for my thoughts.”

Another participant experienced the counselor’s emotional expression as inappropriate after identity disclosure, resulting in feelings of discomfort and a perception of unprofessionalism. Participant G (female, heteroromantic, aged 21) recalled, “When she heard about my specific expressions (of asexuality), she smiled slightly, even chuckled. At that moment, I didn’t feel particularly offended, but surely, it made me somewhat uncomfortable. Afterward, I felt her reaction was actually very unprofessional.”

Regarding evaluations and future intentions, participants who had satisfying experiences generally acknowledged the effectiveness of professional help in providing them with a safe space to openly discuss their pressing concerns and receive assistance. Many expressed their willingness to seek professional help in the future when needed. Among those who had unsatisfactory interactions, two remained optimistic about future help-seeking, and one mentioned considering alternative sources of support. Overall, participants emphasized the need for practitioners to increase their knowledge and understanding of asexuality to enhance services for asexual individuals.

## Discussion

Gaining insights into sexual minority clients’ lives outside therapy sessions is essential to aid therapists in better understanding the dynamics within therapeutic interactions (Israel et al., 2008). The present study is the first to include a thorough exploration of Chinese asexual individuals’ identity-based experiences in relation to their support needs and help-seeking experiences. Accounts from the participants indicated that the provision of effective support to asexual clients requires practitioners to understand the role that the wider sociocultural context plays in influencing clients’ subjective feelings, thoughts, and behaviors. In addition, heterogeneity within the asexual population necessitates practitioners to not make assumptions about their identity, relationships, intimacy needs, and sexuality.

### Understanding Minority Stress Experienced by Chinese Asexual Clients

Participants’ experiences with asexual identity challenges reflect both global asexuality discourse and distinct stressors shaped by Chinese sociocultural values. Compulsory sexuality contributed to their encounters with epistemic denial, dismissive reactions to identity disclosures, and feelings of invisibility. This echoes previous research on how the privileging of sexuality impacts asexual individuals (Cuthbert, 2022; Gupta, 2017a, 2017b). Beyond these influences, participants faced additional layers of minority stress. Some discussed pressure from societal norms and parental expectations to enter into traditional heterosexual marriage and reproduction, aligning with stressors documented among Chinese sexual minority men (Sun et al., 2020). Others expressed fears of being different or concerns about “fitting in,” shaped by collectivistic norms that emphasize conformity (Sun et al., 2020). Additionally, some feared disappointing their parents or changing how others perceived their families if their asexual identity became known, which parallels Ren and Hood’s (2018) discussion on internalized homophobia among Chinese gay men.

The minority stress theory (Meyer, 2003) helps contextualize these findings. External pressures, such as compulsory sexuality, family expectations, and societal norms, function as distal stressors. Internalized concerns, including internalized acephobia, the emphasis on marriage, filial piety, and the belief in continuing the family bloodline, reflect proximal stressors. Recognizing this continuum of stressors can help mental health practitioners understand how broader sociocultural values shape the experiences of Chinese asexual individuals. Although minority stress dynamics are complex and individualized, adopting a broader perspective may assist practitioners to take a more holistic and effective approach in understanding the identity-related challenges of Chinese asexual clients.

### Supporting Asexual Clients’ Identity Exploration

The themes on participants’ journey of exploration toward acknowledging an asexual identity align with previous discussions on asexual individuals’ identity development. Individuals generally experience feelings of alienation and encounter a period of “identity confusion” after they realize they might not be heterosexual (Robbins et al., 2016). Some participants questioned whether they were same-sex attracted or adopted a bisexual label before identifying as asexual, possibly due to the invisibility of asexuality and the assumptions of compulsory sexuality, which prevented them from considering asexuality at that time (Winer et al., 2024). Although this identity confusion period constitutes a normative process of identity development, sexual minority individuals often experience a certain level of distress due to identity uncertainties (Lefevor et al., 2022). Previous research has also suggested that prolonged identity confusion can negatively impact Chinese asexual individual’s mental health (Zheng & Su, 2022). Practitioners have to be aware that for emerging asexual individuals, the lack of visibility and information about asexuality in society can lead them to question themselves about being different, inducing negative feelings (Robbins et al., 2016). They might also adopt identities, including bisexual or pansexual, within the identity exploration journey and sincerely consider themselves to be (Winer et al., 2024). During this stage, it is essential for practitioners to avoid reinforcing compulsory sexuality ideas and provide a safe environment to assist clients in further exploring their sexual identity possibilities.

Participants accessed asexual-related information and interacted with other asexual individuals in online asexual communities that facilitated their identity exploration. This finding is consistent with previous qualitative studies, which found that online communities offer a platform for asexual individuals to explore their identity and access cultural resources (Robbins et al., 2016). Considering the valuable role asexual communities play in participants’ identity exploration, practitioners may consider connecting clients with other asexual individuals where necessary (Foster & Scherrer, 2014). However, it is notable that some participants in the present study mentioned encountering internet negativity in their access to asexual communities, including exposure to cyberbullying, and rumination of ideas—particularly regarding difficulties and challenges in intimate relationships. Practitioners should thus pay attention to clients’ actual experience with online communities even when it is supposed to provide useful resources and support.

### Supporting Asexual Clients’ Identity Negotiation

Participants make sense of their asexual identities in different ways, which supports the argument that asexual individuals in China attach diverse meanings to their identities during the identity negotiation process (Wong & Guo, 2020). Asexual identity has been valuable to many participants as identification allows them to articulate their experiences and contributes to their self-clarification (Carrigan, 2011; Van Houdenhove et al., 2015). However, different from a previous study (Foster & Scherrer, 2014), not all participants perceive asexuality as healthy and advantageous. External factors such as societal attitudes, pathologization, compulsory sexuality assumptions, and cultural-based stigmas can make asexual identification distressing. As an illustration, one participant found being an asexual man shameful, probably because being less “sexual” contradicts traditional notions of masculinity (Tessler & Winer, 2023). Since men are assumed to be sexual and expected to perform sexuality within heterosexuality norms, failing to do so can threaten their status as a “male” (Przybylo, 2014; Winer, 2024a). In this sense, one’s asexuality can be perceived as undermining masculinity (Winer, 2024a), making it unfulfilling for some. However, individuals may also interpret and navigate this experience in diverse ways, shaping their own sense-making of both asexuality and masculinity (Przybylo, 2014; Winer, 2024a).

Practitioners must acknowledge that clients may view their identity differently and avoid taking it for granted. When asexual clients have negative perceptions about their identity and experience related distress, it would be beneficial for the practitioner to acknowledge with clients that these feelings may stem from external influences (Gupta, 2017b) rather than attributing them to clients’ identities or problematizing their asexuality. Under these premises, practitioners may seek out ways to explore clients’ difficulties with their asexual identity in an accepting and culturally sensitive manner, which would hopefully facilitate self-acceptance.

Participants’ decisions to come out involve considerations of the anticipated consequences, particularly the impact of disclosure on relational harmony. This is in line with Chinese lesbians’ coming out concerns, where the emphasis on social harmony in collectivistic values leads to “strong hesitations” regarding identity disclosure (Chow & Cheng, 2010, p. 102). Thus, when asexual clients bring their disclosure concerns, it is crucial for practitioners not to pressure them to disclose without considering their unique situations. If clients make their decisions to disclose, assessing their resources to buffer negative consequences, assisting them in setting up safe disclosure plans, and supporting them in developing coping strategies are considerable ways to provide continual support (Bry et al., 2017).

Participants’ narratives described how engagement in intimate relationships can be challenging. However, some participants mentioned that when asexuality poses challenges to traditional romantic relationships, they negotiate the boundaries of intimacy and develop non-traditional practices of intimacy, such as platonic love or companionships. This echoes the idea that romantic asexual individuals engage in continuous attempts to “negotiate sustainable and satisfying relationships” (Carrigan, 2012, p. 14). To effectively support asexual clients, practitioners should recognize their efforts in pursuing and developing intimacy and affirm their relationships.

The desire or decision to adhere to the traditional norms of sexuality is not uncommon among asexual individuals (Scott & Dawson, 2015). Similar to the participants in the present study, some asexual individuals might seek help from practitioners in hopes of increasing their sexual interest. This requires practitioners to understand that this interest does not necessarily violate their clients’ asexual identity (Gupta, 2017b) and that some asexual individuals can still be “sexual” (Winter-Gray & Hayfield, 2021). Moreover, many participants struggle to navigate societal expectations regarding marriage and offspring. This challenge is not unique to the participants in the present study. An and Kim (2024) noted that societal expectations surrounding marriage and reproduction, along with the normative emphasis on these life stages, compel asexual individuals in South Korea to conform. This similarity reflects the shared influences of Confucianism in both Chinese and South Korean societies, where asexuality challenges traditional social norms. When asexual clients bring related concerns to therapy, practitioners may consider exploring whether their support needs are related to the tension between meeting personal needs and relevant social norms and expectations. Citing Scott and Dawson’s (2015) comment on asexual individuals’ conflict between ideal and conventional relationships, “human nature and social identities are messy, complex and often contradictory; it is common to hold two opposing views simultaneously, and to feel ambivalent towards them” (p. 14). It would be beneficial for practitioners to normalize the dilemma clients encounter, validate their feelings, and assist them in understanding the conflict.

As illustrated by the participants’ identity negotiation process in the present study, Chinese asexual individuals seek out ways to accommodate their asexual identity in the dilemma. Some participants attempt to jump out of the dilemma by challenging dominant societal assumptions, whereas others seek alternative ways to meet social expectations. Practitioners have to keep in mind that asexual individuals may not be passive victims of a pro-sex society; instead, they are “actively and creatively renegotiating the boundaries of the platonic, the intimate and the sexual, with sociocultural ramifications that are potentially far wider than their own community” (Carrigan, 2011, p. 476). The primary role of mental health practitioners is to provide an inclusive environment for them to discuss relevant issues and deliver culturally competent care to support their identity negotiations.

### Advancing Mental Health Care for Asexual Individuals

Negative attitudes and stigma toward professional mental health services serve as barriers to Chinese asexual individuals’ help-seeking, and these barriers apply to Chinese adults as a whole (Shi et al., 2020). As suggested by participants in the current study, launching education and awareness campaigns to destigmatize mental health issues can help to promote mental health literacy. Although help-seeking stigma is not unique to asexual individuals, the pathologization of asexuality and anticipated practitioner biases may further lower asexual individuals’ confidence in mental health help and reduce their help-seeking intentions (Foster & Scherrer, 2014). Depathologizing asexuality and creating an asexual-inclusive environment are important tasks for mental health practitioners to facilitate help-seeking in the asexual population.

Similar to LGBT individuals, asexual individuals in the present study voice concerns regarding confidentiality when seeking help from professionals (McClain et al., 2016). However, regarding provider selection, LGBT individuals often identified helpful providers through referrals from non-professionals (Israel et al., 2008), whereas participants in this study predominantly had practitioners assigned by the agency or selected among the available options provided. To find an asexual-friendly practitioner, some participants mentioned frequently changing counselors, trying different agencies, and raising their budget, which has made their help-seeking more costly. This implies a lack of identifiable providers who work well with members of the Chinese asexual community, further highlighting the pressing need to provide asexual individuals with referral information on supportive providers (Gupta, 2017b).

Participants discussed both satisfying and unsatisfying experiences with mental health practitioners. Firstly, practitioners’ reactions to individuals’ identity disclosure, whether to a “test of water” or a full disclosure, can influence asexual individuals’ willingness to engage in counseling. In line with previous studies (Flanagan & Peters, 2020; Foster & Scherrer, 2014), positive responses involve signaling openness and respect, displaying affirmative attitudes, demonstrating asexuality awareness, and showing the willingness to learn. In contrast, practitioners’ lack of awareness of asexuality, disbelief in one’s identity, and dismissive responses following disclosure were perceived as unhelpful. Incidents of microaggression early in initial conversations about asexuality, including pathologizing assumptions and offensive emotional reactions, can ultimately lead to individuals’ lack of trust in the practitioner and ruptures in the therapeutic alliance (Yeo & Torres-Harding, 2021).

Secondly, practitioners’ demonstration of basic counseling skills is essential to maintain the therapeutic relationship. Participants appreciated their practitioner’s non-judgmental attitudes, acceptance, active listening, empathy, and genuineness. These positive counselor characteristics foster trust building in the relationships, instill a sense of safety in participants, and make them more comfortable discussing their presenting concerns (Flanagan & Peters, 2020). Opposite to these are practitioners’ disengagement, avoidance, coldness, and emotional distance, which were also mentioned among LGBT individuals as unhelpful experiences in psychotherapy (Israel et al., 2008). In the absence of positive counselor characteristics, participants perceived a sense of isolation rather than feeling supported. Lastly, participants noted that counselors’ desire to learn about asexuality enhanced their therapeutic experiences, whereas the lack of interest led to dissatisfaction with the therapeutic process. This is in line with the findings from a previous study, where LGB clients find therapy more beneficial when therapists exhibit humility regarding their sexual orientation (Jennings & Sprankle, 2024).

### Limitations and Further Directions

The present study has several limitations. Firstly, participants were recruited from major online asexual communities in China which has connections to AVEN. Being influenced by the major discourse in communities, participants’ understandings of asexuality are likely to align more closely with the definition presented on AVEN (Hinderliter, 2009). Recruiting participants online has also resulted in a sample that is overrepresented by younger, better-educated students who live in major cities (Ross et al., 2005), which may reduce the generalizability of the findings. Secondly, the recruitment criteria requiring that potential participants attend a minimum of three counseling sessions with the same practitioner may lead to an overrepresentation of positive counseling experiences, as individuals with unsatisfactory experiences would likely have terminated sessions before reaching the third. Further inquiries may explore Chinese asexual individuals’ early termination in mental health settings to gain a comprehensive understanding of their experiences.

Thirdly, the research team of this study did not include a member with specialized expertise in Chinese culture. Nevertheless, both authors are Chinese and are familiar with the Chinese culture. The second author has also conducted numerous studies on multicultural issues in counseling. Recruiting an external expert may provide additional insights to the findings. Additionally, the experiences of individuals within the asexual spectrum are far more diverse than what is presented in the current research due to the small sample size. Future research may examine the experiences of individuals with sub-identities along the asexual and aromantic spectrum. Lastly, this research focuses exclusively on cisgender asexual clients, which overlooks the unique needs faced by asexual individuals with diverse gender identities. Further investigation into how an individual’s asexual and gender identity intersect and shape their help-seeking experiences is also warranted.

### Implications

The above discussion underscores the importance of affirmative practice and culturally competent attitudes, skills, and knowledge when engaging with asexual clients. In addition, cultural humility appears crucial as counselors’ non-judgmental stance and willingness to learn about asexual individuals’ experiences are preconditions of effective counseling. The guideline of cultural humility is especially suitable in working on asexual issues in psychotherapy settings since it requires practitioners’ continual engagement in self-reflection and life-long learning (Tervalon & Murray-García, 1998). To advance mental healthcare for asexual individuals, practitioners are encouraged to engage in critical reflection of their compulsory sexuality assumptions and approach clients’ experiences with respect and humility. The authors hope that the present paper may assist in empowering the voices of asexual individuals in psychotherapy settings, advocating for increased scholarly attention to their support needs and counseling experiences, and fostering future improvements in mental health services.

## Data Availability

Not applicable.

## Appendix: Interview Protocol

### Part I: Asexuality

1. Asexual identity
  - How would you describe your asexuality?
  - How did you come to the conclusion that the asexual identity fits you?
  - How has this identity influenced your life?
2. Asexual communities
  - Have you joined any asexual communities? Why/why not?
  - How has participating in these asexual groups or activities ever affected you?
3. Coming out
  - Have you ever told someone about your asexual identity? Why or why not?
  - How did you experience coming out at that time?
  - How has it influenced your relationships?
  - What is your future disclosure plan?
4. Romantic orientation
  - How would you describe your romantic orientation?
  - How has this orientation affected your relationships?
5. Relationship
  - What is your attitude toward romantic relationships?
  - What does your ideal relationship look like?
6. Physical Intimacy
  - What is your acceptance level of physical intimacy?
  - How do you feel about physical intimacy?
7. Sexuality
  - What is your attitude toward sex?
  - In what circumstance will you consider engaging in sexual behaviors?
8. Marriage and reproduction
  - What is your attitude toward marriage and reproduction?
  - How has your asexual identity influenced your attitude?

### Part II: Psychological Counseling Experience

1. Pre-session
  - Why did you decide to seek psychological counseling this time?
  - What did you expect from the counseling sessions?
  - What are your expectations of the counselor?
  - How is your previous counseling experience?
2. Post-session
  - Would you please describe your counselor?
  - Why did you choose this counselor?
  - How did you tell your counselor about your asexual identity?
  - How did you know about your counselor’s knowledge of asexuality?
  - What was the counselor’s attitude toward your asexuality?
  - How has your counselor been helpful in terms of your asexuality?
  - How has your counselor been helpful in terms of other matters?
  - How is the outcome of the counseling sessions?
  - Why did you terminate/still continue the counseling session?
  - What advice would you give to asexual clients and counselors who work with them?
